# Production of PEGylated Vancomycin-Loaded Niosomes by a Continuous Supercritical CO_2_ Assisted Process

**DOI:** 10.3390/nano14100846

**Published:** 2024-05-13

**Authors:** Lucia Baldino, Domenico Riccardi, Ernesto Reverchon

**Affiliations:** Department of Industrial Engineering, University of Salerno, Via Giovanni Paolo II, 132, 84084 Fisciano, SA, Italy; d.riccardi1@studenti.unisa.it (D.R.); ereverchon@unisa.it (E.R.)

**Keywords:** niosomes, supercritical CO_2_ process, nanovesicles, vancomycin, PEGylation

## Abstract

Niosomes are arousing significant interest thanks to their low cost, high biocompatibility, and negligible toxicity. In this work, a supercritical CO_2_-assisted process was performed at 100 bar and 40 °C to produce niosomes at different Span 80/Tween 80 weight ratios. The formulation of cholesterol and 80:20 Span 80/Tween 80 was selected to encapsulate vancomycin, used as a model active compound, to perform a drug release rate comparison between PEGylated and non-PEGylated niosomes. In both cases, nanometric vesicles were obtained, i.e., 214 ± 59 nm and 254 ± 73 nm for non-PEGylated and PEGylated niosomes, respectively, that were characterized by a high drug encapsulation efficiency (95% for non-PEGylated and 98% for PEGylated niosomes). However, only PEGylated niosomes were able to prolong the vancomycin release time up to 20-fold with respect to untreated drug powder, resulting in a powerful strategy to control the drug release rate.

## 1. Introduction

In recent years, advanced and biocompatible nanocarriers able to improve the active pharmaceutical ingredient bioavailability and ensure its sustained, controlled, and targeted release have been introduced [1,2]. Among the nano-vesicular structures, liposomes and niosomes are attracting considerable interest [3].

Liposomes are formed by phospholipids with one hydrophilic head and two hydrophobic tails that surround an aqueous core [4]. Since this organization mimics that of human cells, these liposomes are characterized by very good biocompatibility when used as drug nanocarriers. However, they are subjected to oxidative degradation [5], could be potentially cytotoxic because of their ionic nature [6], and their costs of production are high because of the expensive raw materials used [7].

With the aim of searching for alternative vesicular formulations that can overcome these disadvantages, niosomes are gathering a significant amount of attention. They are formed by non-ionic surfactants (generally Brij, sodium deoxycholate, Span, Tween) [8,9], which are low cost and biocompatible materials, and can organize themselves in a similar way to liposomes. In particular, niosome formation depends on the hydrophilic–lipophilic balance (HLB) that is a dimensionless parameter and indicates the lipid or water solubility of the surfactant through the balance between its hydrophilic head and hydrophobic tail; HLB can range from 0 to 20, but only values between 4–8 are suitable for forming niosomes [10]. Niosomes are widely used to encapsulate different kinds of molecules and active compounds, such as proteins and peptides [11], vaccines [12,13,14], antibiotics [15], and anticancer drugs [16]. Furthermore, niosomes can be used through different routes of administration, such as intravenous, intramuscular [17], and topical (dermal, ocular, oral, etc.) [18], modifying their properties during the production process and/or adding functional compounds to their structure. In this context, two main kinds of targeting can be obtained: passive targeting, by adding a hydrophilic polymer, like polyethylene glycol (PEG), and leading to the formation of “stealth” niosomes; and active targeting, by functionalizing the membrane surface of niosomes with ligands able to attach to the corresponding receptor expressed on cancer cells [19].

PEG functionalization (i.e., PEGylation) is one of the most used strategies to obtain targeted drug delivery [20] because of the polymer biocompatibility and low toxicity [21,22]. The key role of PEG on the niosome surface is to attract water molecules thanks to its hydrophilic nature, obtaining a water shell around the nanovesicles. This conformation can lead to the generation of “stealth” niosomes because of the reduced uptake and blood clearance of the nanovesicles by the reticuloendothelial system [23,24]. Cosco et al. [25] produced PEGylated niosomes via the thin-layer evaporation technique, and encapsulated 5-fluoracil, an antitumoral compound used for breast cancer treatment. They obtained loaded niosomes of about 480 nm and, after a sonication step, the dimensions were reduced to 230 nm; PEGylated niosomes presented a slightly larger diameter (500 nm and 250 nm, before and after sonication step, respectively) and a drug encapsulation efficiency of 50%. Kopermsub et al. [26] prepared nisin and ethylenediamine tetra-acetic acid (EDTA)-loaded niosomes using 80 mM of Span 80, 0.1, 0.5, 1 mM of sodium stearoyl lactate (SSL), an anionic surfactant, and PEG400 (1% *w*/*v*). After 12 repeated extrusions throughout a polycarbonate membrane, niosomes with a mean diameter of about 200 nm and an encapsulation efficiency ranging from 5% to 29% for the best formulation were obtained. Baranei et al. [27] prepared niosomes loaded with green tea extracts using Tween 40 (10 mM), Span 40 (10 mM), cholesterol, and PEG3000 (2% *w*/*v*) using the thin-film hydration method. Sonication and extrusion throughout the membrane were performed to obtain small and homogeneous unilamellar nanovesicles; non-PEGylated and PEGylated niosomes of about 150 nm and 240 nm, respectively, were obtained and an 80% encapsulation efficiency was reached in the case of PEGylated nanovesicles.

In all of these works, conventional and multi-step techniques were used to produce niosomes that were generally characterized by large mean diameters and size distributions, and low drug encapsulation efficiency; moreover, post-processing steps were used to reduce their dimensions. These disadvantages can be overcome using an innovative and continuous process assisted by supercritical CO_2_ (SuperSomes) demonstrated to successfully produce nanometric liposomes [28,29,30], transfersomes [31], and niosomes [32,33] at high drug encapsulation efficiency and unimodal particle size distribution, without post-processing operations [30].

Therefore, in this work, SuperSomes was used to produce niosomes starting from different formulations in which the weight ratio between Span 80/Tween 80 and the presence of cholesterol were varied to investigate the effect of HLB parameter on the nanovesicle formation. Subsequently, vancomycin, an antibiotic used against Gram-positive bacteria [34], was selected as a model active compound to study the effect of PEGylation on the drug release rate.

## 2. Materials and Methods

### 2.1. Materials

Span 80 (M_w_ = 428.60 g/mol; HLB = 4.3), Tween 80 (M_w_ = 1310 g/mol; HLB = 15), cholesterol powder (M_w_ = 386.6 g/mol, purity > 99%), polyethylene glycol 400 (PEG400), and vancomycin (M_w_ = 1449.3 g/mol) were purchased from Sigma Aldrich (Milan, Italy). Ethanol (anhydrous, ≥99.9%) was purchased from Carlo Erba Reagents (Cornaredo (MI), Italy). Distilled water was produced in situ using a lab-scale distiller. Carbon dioxide (CO_2_, >99.4%) was purchased from Morlando Group Srl (Naples, Italy).

### 2.2. SuperSomes Process and Plant Description

Three lines, water solution, ethanolic solution, and CO_2_, are fed to a high-pressure vessel where the vesicles are formed. CO_2_ is refrigerated at −10 °C using a cryostat (Julabo mod. FL300, Milan, Italy) to reach the liquid state; then, it is pumped to a stainless-steel saturator (internal volume 0.15 dm^3^, filled with stainless-steel Berls saddles, 1889 m^−1^ speficic surface, 0.94 voidage, 0.16 in., Pro-Pak, Scientific Development Company, State College, PA, USA) using an EcoFlow^®^ pump (mod. LDC-M-2, Lewa, Leonberg, Germany). Ethanol solution (100 mL) is pumped to the same saturator using a Gilson pump (mod. 305, Villiers Le Bel, France) at a gas-to-liquid ratio (GLR) equal to 2.40 (*w*/*w*). The obtained gas-expanded liquid (CO_2_ + ethanol) is sent to the high-pressure stainless-steel vessel (internal volume 0.50 dm^3^) throughout a capillary tube, whereas water solution is atomized throughout a micrometric injector (80 μm diameter). CO_2_ and ethanol are sent during the process to a separator though a micrometering valve (mod. 1335G4Y, Hoke, Spartanburg, SC, USA). This separator is operated at 25 °C and 10 bar; therefore, a separation between gaseous CO_2_ and ethanol is obtained. In the whole process, pressure is controlled using a pressure gauge (mod. MP1, OMET, Lecco, Italy) and temperature is measured by type J thermocouples and controlled using PID controllers (Series 93, Watlow, Corsico (MI), Italy). The niosomal suspension is collected in a 0.16 dm^3^ stainless-steel reservoir and recovered by opening an on/off valve (mod. SS-43GS4, Swagelok, Solon, OH, USA). Other details about the plant description can be found in previous papers [28,32,33].

### 2.3. Characterization Analyses

Nanovesicles were characterized in terms of mean hydrodynamic diameter (MHD), ζ-potential, morphology, and drug encapsulation efficiency. Measures of MHD and ζ-potential were performed in triplicate using dynamic light scattering (DLS, mod. Zetasizer Nano S, Worcestershire, UK).

The niosomes’ morphology was observed by a field emission scanning electron microscope (FE-SEM, mod. LEO 1525, Carl Zeiss SMTAG, Oberkochen, Germany). Preparation of the samples for FE-SEM analysis consisted of depositing a small volume of suspension on an aluminum stub; after air-drying for two days, the stub was covered by a thin gold film layer using a sputter coater (mod. 108 A, Agar Auto Sputter Coater, Stansted, UK) at 40 mA for 150 s.

The vancomycin encapsulation efficiency was obtained by measuring the free active compound content in the supernatant. The niosome suspension was first centrifuged at 32,000 rpm for 60 min at 4 °C (mod. Optima XE-100 IVD, equipped with a Type-32 Swinging Bucket Rotor, Beckmann Coulter, Cassina De’ Pecchi (MI), Italy) and the recovered supernatant was analyzed using a UV-Vis spectrophotometer (mod. Cary 60, UV-vis, Agilent Technologies, Santa Clara, CA, USA), reading the vancomycin absorbance at λ = 280 nm [35]. Finally, the encapsulation efficiency was calculated using Equation (1) [32]:

EE% = (1 − supernatant concentration/theoretical concentration) × 100(1)

where the theoretical concentration was the concentration of the drug dissolved in the starting solution, and the supernatant concentration was the concentration obtained by reading the supernatant absorbance.

In vitro drug release tests were performed according to the following procedure: 5 mL of niosomal suspension was inserted in a 14,000 Da cut-off dialysis sack (Sigma Aldrich, Milan, Italy); the dialysis sack was sealed and immersed in 80 mL of phosphate-buffered saline (PBS, pH = 7.4) at 37 °C and continuously stirred at 250 rpm. The vancomycin absorbance was read at different time intervals, at λ = 280 nm, using the UV-Vis spectrophotometer described before.

## 3. Results and Discussion

All experiments were performed using SuperSomes apparatus at 100 bar and 40 °C. The water flow rate was set at 7 mL/min and the ethanolic flow rate at 3.5 mL/min [31,32].

### 3.1. Production of Unloaded Niosomes

In the first series of experiments, Span 80/Tween 80 weight ratio was varied with the aim of comparing the mean diameter and morphology of the obtained vesicles (total surfactant amount equal to 2000 mg). Three different ratios of surfactant were investigated, and the obtained formulations are reported in Table 1.

The Nio01, Nio02, and Nio03 samples were characterized by an MHD of 181 ± 55 nm, 191 ± 47 nm, and 248 ± 52 nm, and ζ-potential values equal to −16.2 ± 2.5 mV, −19.8 ± 2.5 mV, and −23.1 ± 2.3 mV, respectively. When the values of HLB increased, the niosomes’ mean size became larger. This result agrees with those of other studies [36,37] and can be explained by the higher surface energy and the more hydrophilic structure of the nanovesicles when prepared with a larger amount of Tween 80. This feature led to a higher uptake of water in the vesicle inner core, producing larger vesicles [38]. However, the effect of HLB on the ζ-potential of the suspensions produced was not relevant, since the values obtained did not present a remarkable difference [36]; in particular, stable nanovesicles were expected since these values (in modulus) of ζ-potential indicated high electrostatic repulsive forces among nanoparticles, because of the same superficial charge [39,40]. Stability measurements at regular time intervals (0, 15, 30, and 60 days from the production) were thus performed by DLS on nanovesicles stored at 4 °C. The results are summarized in Table 2.

Generally speaking, slight variations in the MHD and ζ-potential of niosomes after 60 days from production emerged.

**Figure 1:**
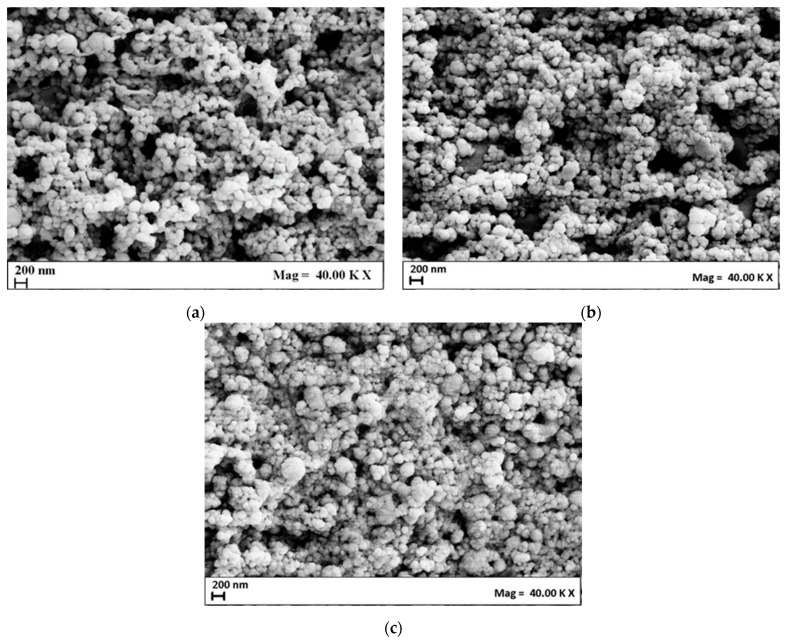
FE-SEM images of Nio01 (**a**), Nio02 (**b**), and Nio03 (**c**) samples.

The morphology of the niosomes was investigated by FE-SEM analysis and example images are presented in Figure 1. The niosomes showed a spherical morphology, whose mean diameter was in line with the one obtained by DLS analysis. The presence of aggregates in the SEM images was due to coalescence phenomena induced by the air-drying step during sample preparation before the analysis [31,32,33].

Subsequently, the effect of cholesterol loading in the niosomal bilayer on the mean diameter and morphology of vesicles was investigated. In particular, the literature suggested to add cholesterol to lipid-based formulations with an HLB larger than 6 [8,41]; for this reason, it was added to the formulation presenting an HLB equal to 6.44 (i.e., Nio04 with a Span 80/Tween 80 equal to 80:20% *w*/*w*), using a surfactant/cholesterol molar ratio equal to 4 [42].

In this case, nanovesicles with a mean diameter of 118 ± 33 nm and a ζ-potential equal to −27.4 ± 3.0 mV were produced. Comparing samples without (Nio03) and with (Nio04) cholesterol, at the same HLB value, it is possible to observe that cholesterol influenced the vesicle mean diameter: in particular, MHD was lower when cholesterol was added to the starting formulation, because it can enhance the bilayer hydrophobicity; therefore, the vesicle surface energy decreased, leading to the formation of smaller niosomes [42]. Moreover, the ζ-potential was higher in the modulus (from −23.1 mV to −27.4 mV), highlighting that a more stable structure was produced. Indeed, the interaction between the hydroxyl group of cholesterol and the alkyl chain of surfactants, by means of a hydrogen bond, increases the transition temperature of the nanovesicles, leading to a more stable structure [8].

Stability analysis over time was also performed in this case at regular time intervals (0, 15, 30, 60 days from production), stocking the samples at 4 °C. The results (see Table 2) confirmed a negligible increase in the niosomes’ mean diameter after 60 days of monitoring.

**Figure 2:**
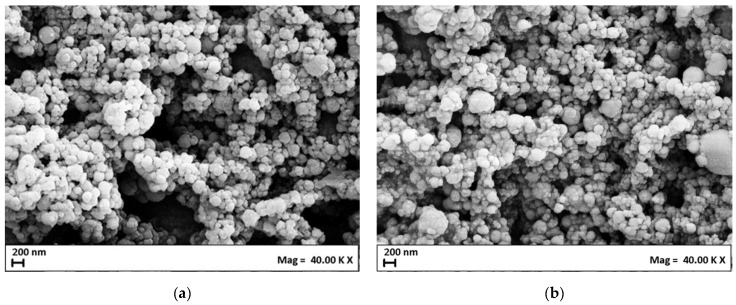
FE-SEM images of Nio04 (**a**) and Nio05 (**b**) samples.

The FE-SEM images in Figure 2a confirm the spherical shape of these niosomes, indicating that cholesterol did not influence the morphology and shape of the nanovesicles.

Since the niosomes from the Nio04 experiments showed positive results in terms of mean diameter, stability over time, and ζ-potential, they were further investigated by adding PEG and evaluating its influence on the characteristics of the “stealth” nanovesicles. PEG400 was chosen as the coating agent, according to a previous study by Javani et al. [43], where different kinds of PEG were investigated (PEG400, PEG1500, and PEG10000), and PEG400 showed the formation of smaller niosomes that were stable over time, and with the highest drug encapsulation efficiency.

PEG400 was added to the water solution (1% *w*/*v*) and processed by SuperSomes at the same operating conditions used for the production of non-PEGylated niosomes.

Niosomes with a mean diameter of about 202 ± 61 nm and a ζ-potential of −30.6 ± 2.4 mV were obtained. They showed a larger mean diameter than non-PEGylated vesicles, in accordance with other literature studies [25,26,44,45]. This trend is due to the presence of PEG that interacts with surfactant molecules by hydrogen bonding, altering the packing of the bilayer membrane. This effect produces an increase in the curvature radius of the nanovesicles and, therefore, larger vesicles are obtained [43,45]. Moreover, the ζ-potential of PEGylated nanovesicles was higher in the modulus than that of non-PEGylated nanovesicles; this behavior can be attributed to the presence of the PEG coating [46,47] that creates a steric barrier on the niosomal surface, able to contrast aggregation phenomena among vesicles [48].

Also, the PEGylated niosomes maintained a constant mean diameter and ζ-potential up to 60 days from production (see Table 2); moreover, their morphology remained spherical despite PEG400 being added to the formulation, as shown in Figure 2b.

### 3.2. Production of Vancomycin-Loaded Niosomes

In the last part of the work, niosomes loaded with an active compound were produced. Considering the promising results obtained from the first part of the experimentation, the formulation with a Span 80/Tween 80 weight ratio equal to 80:20, loaded with cholesterol, was selected for the encapsulation of vancomycin (5% *w*/*w* drug-to-lipid ratio). Furthermore, PEG400 was also added to this formulation to determine the overall effect on the niosomes’ size and size distribution, ζ-potential, stability over time, morphology, encapsulation efficiency, and drug release rate. The tested formulations are reported in Table 3.

**Figure 3:**
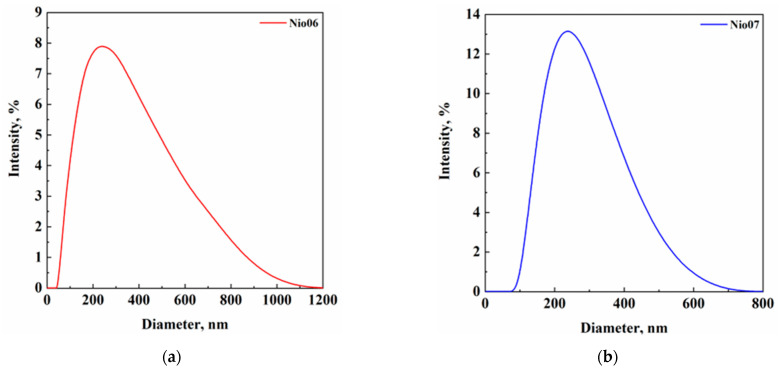
Vesicle size distribution of (**a**) non-PEGylated (Nio06) and (**b**) PEGylated (Nio07) vancomycin-loaded niosomes.

In this set of experiments, nanometric niosomes with a unimodal size distribution (Figure 3) and high in modulus ζ-potential values (−31.1 ± 2.5 mV and −27.7 ± 2.0 mV for non-PEGylated and PEGylated niosomes, respectively) were obtained. Specifically, they were characterized by a mean diameter equal to 214 ± 59 nm and 254 ± 73 nm for non-PEGylated and PEGylated niosomes, respectively. The diameters were larger with respect to the ones measured for unloaded niosomes and this result may be due to the active compound encapsulation.

The niosomes’ stability after 0, 15, 30, and 60 days from production was investigated, and the results are summarized in Table 4.

**Figure 4:**
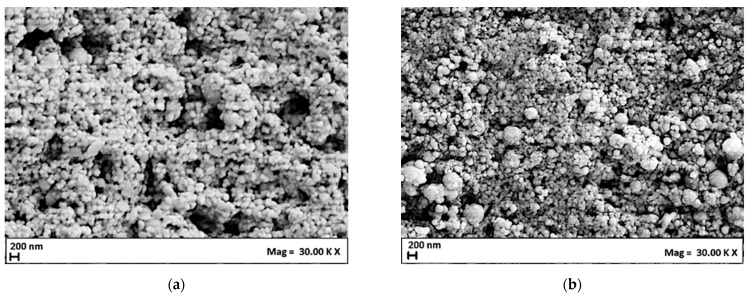
FE-SEM images of Nio06 (**a**) and Nio07 (**b**) samples.

Drug-loaded non-PEGylated and PEGylated niosomes were stable over time and their morphology (Figure 4a,b) was spherical, demonstrating that the addition of a drug did not influence the physical properties of these nanovesicles.

UV-Vis spectrophotometer analysis was performed to measure the drug encapsulation efficiency and to investigate the effect of the PEG coating on the vancomycin release rate. The encapsulation efficiency calculated for non-PEGylated niosomes was equal to 95%, whereas it was equal to 98% for the PEGylated ones. Although the vancomycin encapsulation efficiency was over 90% in both niosomal preparations, the PEGylated niosomes presented superior performance, as reported in the literature [23,49,50]; i.e., PEG insertion between surfactant molecules tends to create a more compact and condensed structure during the bilayer formation phase, resulting in better final drug encapsulation efficiency [51]. Moreover, this evidence could be the result of the SuperSomes process since, after the atomization of the water phase containing vancomycin, droplets were quickly surrounded by a first layer of surfactants, forming reverse micelles and entrapping the active compound in the aqueous core [28].

**Figure 5:**
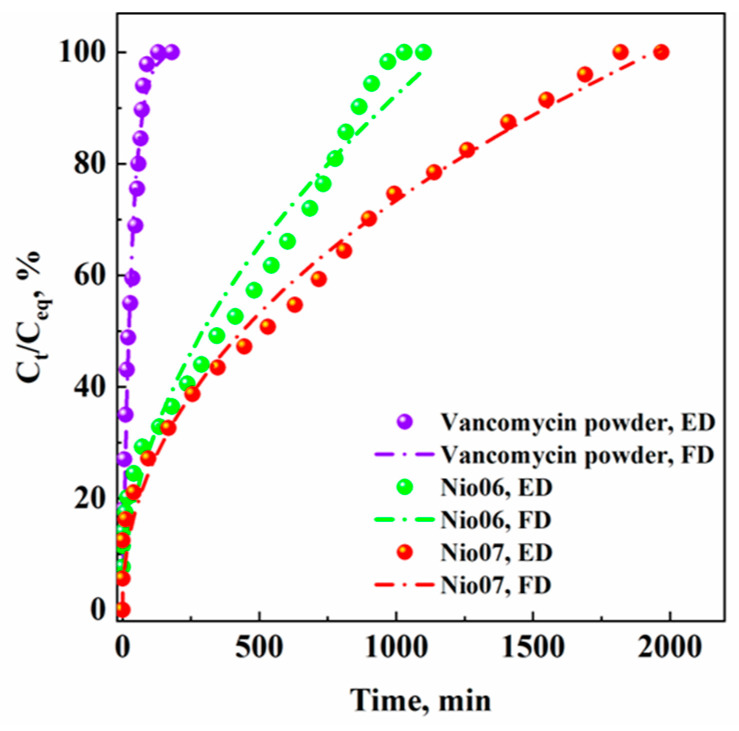
Release profiles of vancomycin powder, Nio06, and Nio07 samples. ED: experimental data; FD: fitted data.

The release profiles of the vancomycin powder, Nio06, and Nio07 samples are shown in Figure 5; they are reported in terms of percentage ratio between the drug concentration measured at different time intervals (C_t_) and the maximum drug concentration released (C_eq_) in the liquid medium versus time (min).

Figure 5 shows that the vancomycin dissolved in PBS in 90 min. The vancomycin loaded in niosomes prepared with cholesterol (Nio06) was released in about 1000 min, whereas the vancomycin loaded in PEGylated niosomes prepared with cholesterol (Nio07) was released in about 1800 min. This result demonstrates that PEGylation strongly influenced the drug release kinetics, since the PEG-coating increases the resistance associated with the active compound diffusion from the inner core to the outside of the nanovesicle [52,53].

Vancomycin release profiles were then fitted with three mathematical models generally used for non-linear active compound diffusion profiles [33,54,55]: first order (Equation (2)), Korsmeyer–Peppas (Equation (3)), and Higuchi (Equation (4)). The adjusted R^2^ coefficient (R^2^_adj_) and the Akaike Information Criterion (AIC) were used to verify which model properly fitted the experimental data.

C_t_/C_∞_ = a·(1 − exp(−b·t)), (2)

C_t_/C_∞_ = k_KP_·t^n^, (3)

C_t_/C_∞_ = k_H_·t^1/2^, (4)

where C_t_ and C_∞_ represent the concentration of vancomycin released at time “t” and at infinite time, respectively; a, b, n, k_KP_, and k_H_ are the constant parameters of each model. In Table 5, the values of the parameters, R^2^_adj_ and AIC, are reported and the equation that returned the largest R^2^_adj_ and the lowest AIC coefficient was selected as the best-fitting model.

The first-order model was the best-fitting model for the vancomycin powder release profile, since the value of R^2^_adj_ and AIC were the higher and the lower ones, respectively, among those calculated and reported in Table 5. The best kinetic model to fit the drug release from non-PEGylated niosomes (Nio06) was the Higuchi model that is based on Fickian diffusion [56]. Lastly, the experimental data of drug release from PEGylated niosomes (Nio07) were properly fitted by the Korsmeyer–Peppas model. In particular, the value of the exponent “n” in the Korsmeyer–Peppas equation can explain what kind of diffusion mechanism is involved during the drug release; in this case, since n was equal to 0.47, an anomalous and non-Fickian diffusion mechanism was involved [57], probably due to the presence of PEG in the niosome structure.

## 4. Conclusions

In this work, different surfactant ratios (Span 80/Tween 80) for the production of niosomes via a supercritical CO_2_-assisted process were investigated. In all cases, stable nanovesicles, with a unimodal size distribution, were obtained. When cholesterol was added to the formulation with a HLB higher than 6, niosomes characterized by a smaller mean diameter were produced due to the vesicles’ surface energy decrease.

Non-PEGylated and PEGylated vancomycin-loaded nanoniosomes, characterized by a unimodal particle size distribution and stability over time, were also successfully produced by SuperSomes. In particular, PEGylated niosomes showed a larger active compound entrapment with respect to the non-PEGylated ones, as a consequence of the polymer coating that led to a more rigid structure. Vancomycin release profiles demonstrated that the encapsulation strategy prolonged the drug release by about 11 times compared with the free vancomycin powder, whereas PEGylation increased the drug release time by about 20 times in comparison with pristine vancomycin, following an anomalous and non-Fickian diffusion mechanism. Therefore, PEG addition favored the formation of a more compact structure, characterized by a larger mass resistance to the drug diffusion, and resulting in a controlled and delayed release.

## Figures and Tables

**Table 1:** Formulations tested for niosome production by SuperSomes (HLB of the mixture was calculated according to the equation reported in previous studies [32,33]).

Set of Experiments	Span 80/Tween 80	HLB	Span 80, mg	Tween 80, mg	Surfactant/Cholesterol, mol/mol	PEG400, % *w*/*v*
Nio01	95:5	4.84	1900	100	-	-
Nio02	90:10	5.37	1800	200	-	-
Nio03	80:20	6.44	1600	400	-	-
Nio04	80:20	6.44	1600	400	4	-
Nio05	80:20	6.44	1600	400	4	1

**Table 2:** Stability results of unloaded niosomes stored at 4 °C up to 60 days from production.

Sample	Stability, Days	MHD, nm	PDI	ζ-Potential, mV
Nio01	0	181 ± 55	0.365	−16.2 ± 2.5
	15	180 ± 49	0.297	−16.1 ± 3.6
	30	181 ± 54	0.362	−17.1 ± 2.7
	60	185 ± 51	0.301	−15.0 ± 2.2
Nio02	0	191 ± 47	0.242	−19.8 ± 2.5
	15	190 ± 50	0.276	−19.8 ± 3.1
	30	188 ± 52	0.302	−17.4 ± 3.0
	60	189 ± 56	0.348	−15.3 ± 2.0
Nio03	0	248 ± 52	0.182	−23.1 ± 2.3
	15	248 ± 58	0.222	−21.0 ± 2.7
	30	247 ± 58	0.228	−19.7 ± 2.4
	60	245 ± 56	0.210	−18.5 ± 2.5
Nio04	0	118 ± 33	0.318	−27.4 ± 3.0
	15	118 ± 34	0.325	−27.4 ± 3.0
	30	118 ± 35	0.357	−25.7 ± 4.0
	60	119 ± 39	0.424	−23.1 ± 3.1
Nio05	0	203 ± 62	0.372	−30.6 ± 2.4
	15	203 ± 62	0.372	−30.6 ± 2.4
	30	204 ± 63	0.381	−30.7 ± 2.4
	60	205 ± 67	0.430	−30.8 ± 2.7

**Table 3:** Formulations tested for the production of vancomycin-loaded niosomes by SuperSomes.

Set of Experiments	Span 80/Tween 80	Span 80, mg	Tween 80, mg	Surfactant/Cholesterol, mol/mol	PEG400, % *w*/*v*	Drug to Lipid Ratio, % *w*/*w*
Nio06	80:20	1600	400	4	-	5
Nio07	80:20	1600	400	4	1	5

**Table 4:** Stability results of vancomycin-loaded non-PEGylated and PEGylated niosomes.

Sample	Stability, Days	MHD, nm	PDI	ζ-Potential, mV
Nio06	0	214 ± 59	0.300	−31.1 ± 2.5
	15	214 ± 57	0.300	−31.1 ± 2.5
	30	214 ± 65	0.372	−31.1 ± 2.3
	60	214 ± 65	0.370	−31.0 ± 2.0
Nio07	0	254 ± 73	0.330	−27.7 ± 2.0
	15	254 ± 73	0.330	−27.7 ± 2.0
	30	250 ± 66	0.280	−27.6 ± 2.4
	60	250 ± 65	0.270	−27.3 ± 2.0

**Table 5:** Parameters obtained by fitting the experimental data of vancomycin release profile, using different mathematical models.

		Vancomycin Powder	Nio06	Nio07
First order	a	100.18	118.3	103.42
	b	0.0298	0.0016	0.0014
	R^2^_adj_	0.965	0.921	0.941
	AIC	70.05	118.83	98.71
Korsmeyer–Peppas	k_KP_	15.78	2.40	2.95
	n	0.38	0.53	0.47
	R^2^_adj_	0.950	0.960	0.985
	AIC	70.76	95.61	62.68
Higuchi	k_H_	9.57	2.92	2.31
	R^2^_adj_	0.899	0.961	0.983
	AIC	79.54	93.55	62.74

## Data Availability

All data are contained within the article.
